# On-chip coherent detection with quantum limited sensitivity

**DOI:** 10.1038/s41598-017-05142-1

**Published:** 2017-07-06

**Authors:** Vadim Kovalyuk, Simone Ferrari, Oliver Kahl, Alexander Semenov, Michael Shcherbatenko, Yury Lobanov, Roman Ozhegov, Alexander Korneev, Nataliya Kaurova, Boris Voronov, Wolfram Pernice, Gregory Gol’tsman

**Affiliations:** 1 Department of Physics, Moscow State Pedagogical University, Moscow, 119992 Russia; 2 Institute of Nanotechnology, Karlsruhe Institute of Technology, Karlsruhe, 76132 Germany; 3 Institute of Physics, University of Münster, Münster, 48149 Germany; 4 Moscow Institute of Physics and Technology, Moscow, 141700 Russia; 5 National Research University Higher School of Economics, Moscow, 101000 Russia

## Abstract

While single photon detectors provide superior intensity sensitivity, spectral resolution is usually lost after the detection event. Yet for applications in low signal infrared spectroscopy recovering information about the photon’s frequency contributions is essential. Here we use highly efficient waveguide integrated superconducting single-photon detectors for on-chip coherent detection. In a single nanophotonic device, we demonstrate both single-photon counting with up to 86% on-chip detection efficiency, as well as heterodyne coherent detection with spectral resolution *f/∆f* exceeding 10^11^. By mixing a local oscillator with the single photon signal field, we observe frequency modulation at the intermediate frequency with ultra-low local oscillator power in the femto-Watt range. By optimizing the nanowire geometry and the working parameters of the detection scheme, we reach quantum-limited sensitivity. Our approach enables to realize matrix integrated heterodyne nanophotonic devices in the C-band wavelength range, for classical and quantum optics applications where single-photon counting as well as high spectral resolution are required simultaneously.

## Introduction

Nanophotonic circuits allow for realizing complex optical functionality on a chip and enable the assembly of functional devices with many optical components in a scalable fashion. Fast and efficient single-photon detectors represent one of the fundamental building blocks for the realization of on-chip quantum photonic circuits^1^. Superconducting nanowire single-photon detectors^2^ (SNSPDs) are the most promising detection devices for telecommunication wavelengths. They combine high detection efficiency, low dark count rate, and high temporal resolution in a single device^3, 4^ and have been successfully employed for classical and quantum optics applications^5^. In contrast to a more traditional detector geometry, in which light illuminates the nanowire surface under normal incidence, a travelling wave approach^6^ allows drastically increased absorption efficiency due to evanescent coupling with a waveguided mode, resulting in a compact device which can be embedded within large-scale quantum photonic integrated circuits^7^. By adapting the length and the width of the nanowire, one can precisely control the detector absorption and the internal detection efficiency (IDE). This enables the realization of detectors with over 90% on-chip detection efficiency (*η*_*oc*_) in the C-band^8^. To date, such hybrid superconducting-nanophotonic devices have been implemented on a variety of material platforms^9^, including silicon on insulator (SOI), silicon nitride (Si_3_N_4_), gallium arsenide (GaAs) and polycrystalline diamond. The latest success of the traveling wave approach is associated with the detection of non-classical light on a chip^10, 11^.

As threshold detectors, SNSPDs are not directly capable of giving information about the energy of the incident radiation. Integrated optics offers possible solutions, introducing additional elements such as arrayed waveguide gratings (AWG)^12^, Mach-Zehnder interferometers^13^, ring resonators^14^, extended waveguides^15^, diffraction gratings on planar waveguides^16^ and photonic crystals^17^. However, all of these approaches offer only a small number of spectral channels, which results either in limited resolution or in a narrow spectral range.

An alternative way of determining the spectrum of unknown radiation is to use coherent detection^18^. With this technique, the detector absorbs radiation from two different sources (signal S and local oscillator LO) which are frequency-shifted relatively to each other. The response signal is a difference (intermediate) frequency (IF), corresponding to the beating frequency of the two fields. Knowing the LO frequency and measuring the IF allows to determine the frequency and phase of the unknown signal S.

While analog detectors are normally used for coherent detection, recently, the use of a Geiger-mode operated InGaAs avalanche photodiode (APD) array for heterodyne detection at 1064 nm wavelength has been demonstrated in a single-photon counting regime^19, 20^. This approach allows to combine the detection of photons as well as heterodyne mixing and is of interest for Doppler shift detection of specular and diffused targets during LADAR investigation. Furthermore, using photon counters in the coherent detection allows to reduce LO power by more than 9 orders of magnitude from milli- to picowatts level. Potential of SNSPDs for coherent detection at telecommunication wavelength was introduced by Shcherbatenko *et al*.^21^, where he demonstrated that a single pixel SNSPD is capable of taking over the entire functionality of an APD array, with LO power required of the order of a few pW and providing IF bandwidth up to 140 MHz. However, due to limited detection efficiency and nonscalability, the application of stand-alone SNSPDs for coherent detection is substantially limited.

Combining this technique and the superior sensitivity of SNSPDs on an integrated platform with high *η*_*oc*_ allows us to carry out on-chip heterodyne detection with high spectral resolution. We employ waveguide-integrated SNSPDs for on-chip coherent detection and demonstrate quantum limited sensitivity with a chip-based device. Our approach provides advantages not only for Doppler shift detection^19, 20^ and weak signal frequency modulation^22^, but also for integrated quantum optics technologies in the context of correlation and spectral characterization of on-chip narrowband single-photon sources^23, 24^.

## Results

**Figure 1:**
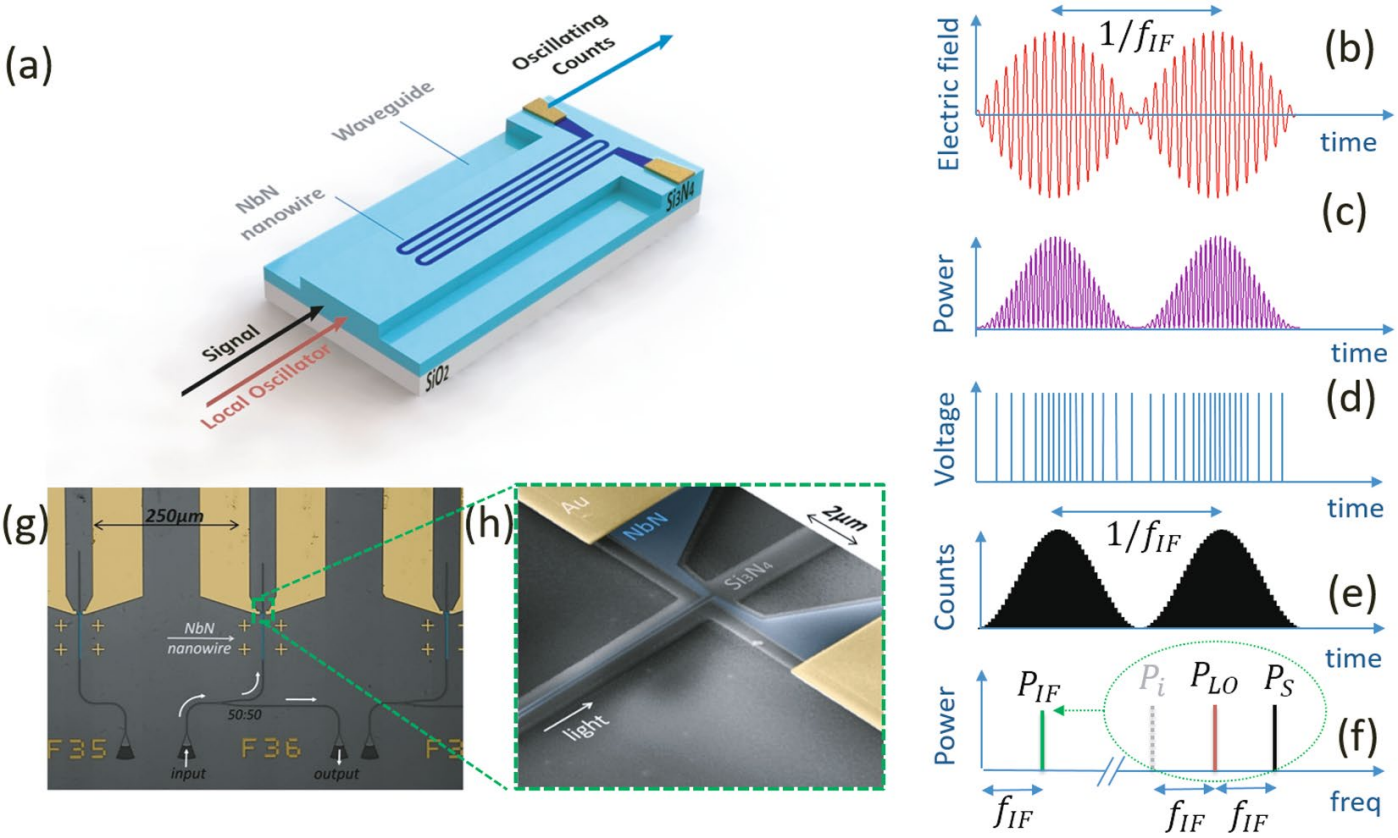
Operation principle of a SNSPD as a coherent detector and layout. (**а**) Schematic view of the SNSPD in coherent detection mode, in which two EM waves with slightly different frequencies (*f*_*LO*_, *f*_*S*_) are sent to the SNSPD. (**b**) Time domain beating of the EM-field generated overlapping LO and S fields, with slightly different frequencies. (**с**) Power oscillation in time at an intermediate frequency *f*_*IF*_ = |*f*_*LO*_ − *f*_*S*_|. (**d**) Schematic representation of the detection pulses frequency modulation in presence of beating, which reproduces the optical power amplitude modulation. (**e**) Schematic view of the counts histogram vs time. By measuring the frequency of the counts oscillations *f*_*IF*_ and knowing the *f*_*LO*_, one can determine the frequency of the unknown signal *f*_*S*_ = |*f*_*LO*_ − *f*_*IF*_|. (**f**) Representation of the coherent detection in the frequency domain. The intermediate frequency is transferred from the IR range to a lower frequency. In addition to the signal and LO, the image channel contributes noise to *P*_*IF*_. (**g**) False color optical micrograph of the nanophotonic devices. Each device consists of two focusing grating couplers, nanophotonic waveguides with a 50:50 Y-splitter, where one branch reaches a NbN nanowire connected to Au contact pads. (**h**) False color SEM image of a typical NbN nanowire atop a nanophotonic waveguide.

The adopted coherent detection scheme requires two radiation sources, a signal (S) and a local oscillator (LO), with slightly different frequencies *f*_*LO*_, *f*_*S*_ (Fig. 1a). The superposition of the two waves generates a beating signal with an amplitude which rises when the two signals are in phase and falls when in anti-phase (Fig. 1b). The beating power, defined as ${P}_{IF}=2\sqrt{{P}_{S}{P}_{LO}}\,\cos (2\pi {f}_{IF}t+\phi )$, oscillates at an intermediate frequency given by the difference of the two lasing frequencies: *f*_*IF*_ = *f*_*LO*_ − *f*_*S*_ (Fig. 1с). As a consequence of this beating mode also the photon flux (Ф_*ph*_), and therefore, the count rate (CR) from the detector oscillates at the same frequency *f*_*IF*_ (Fig. 1d,e). While the timestamp of each detection event is non-deterministic, the overall response probability is proportional to the incoming power in each time bin. In the presence of beating, the response probability is: *p*(*t*) = Φ_*ph*_ *η*_*oc*_(1 + *α* cos(2*π f*_*IF*_ *t* + *φ*)), where *α* is the modulation depth $\alpha =2\sqrt{{P}_{S}{P}_{LO}}/({P}_{S}+{P}_{LO})$. Knowing the LO frequency and measuring the intermediate frequency, it is then possible to determine the frequency of the unknown signal *f*_*S*_ = |*f*_*LO*_ − *f*_*IF*_| as well is its amplitude *P*_*S*_ ∝ *P*_*IF*_^2^/*P*_*LO*_.

Two different methods can be adopted to investigate the signal with a SNSPD in coherent detection mode. A first approach consists in registering the arrival time of the detection pulses in a pulse counter. The intermediate frequency is then *f*_*IF*_ = 1/*T*_*beat*_, where *T*_*beat*_ is the period of the beating (Fig. 1e). A second approach consists in spectrally resolving the detection pulses train using a spectrum analyzer (SA), which converts the signal into the frequency domain providing power spectral density (PSD) of the signal (Fig. 1f). In practice, the integration time is determined by the desired frequency resolution and the frequency range in which the measurement is made. The stability of the LO determines the maximum frequency resolution, and the detector conversion bandwidth determines the frequency window in which measurements are usually made. In turn, the accumulation efficiency (signal-to-noise ratio) is proportional to the detection efficiency of the detector and limited by the detector and system noise. In order to increase the signal-to-noise ratio (SNR), sequences of the data accumulation steps could be repeated providing an averaged result, with the increase of the total measurement time. For this reason, efficient, low-noise and broadband detectors are desirable.

### Device design

Color-enhanced optical and SEM pictures of the fabricated nanophotonic circuit are shown in Fig. 1g,h. The device structure includes two focusing grating couplers (FGC), a 50:50 Y-splitter and a nominal 4 nm thick NbN nanowire on top of the silicon nitride rib waveguide. For our experiment, we use SNSPDs with the same nanowire width (80 nm), but different length and shape. In particular, we use a W - shaped nanowire (WSN), 240 µm long, represented in Fig. 1a, and a U – shaped nanowire (USN) 140 µm long, depicted in Fig. 1h. The FGCs are used to inject light into a single mode nanophotonic waveguide and are also used for the collection of the transmitted light from the chip. The period and the fill factor of the FGCs are optimized for transmission at 1550 nm wavelength at liquid helium temperatures. The 50:50 Y-splitter is employed to route equal shares of the light to the SNSPD and to a second FGC, which serves as a reference to determine the photon flux reaching the detector.

### Single-photon counting

**Figure 2:**
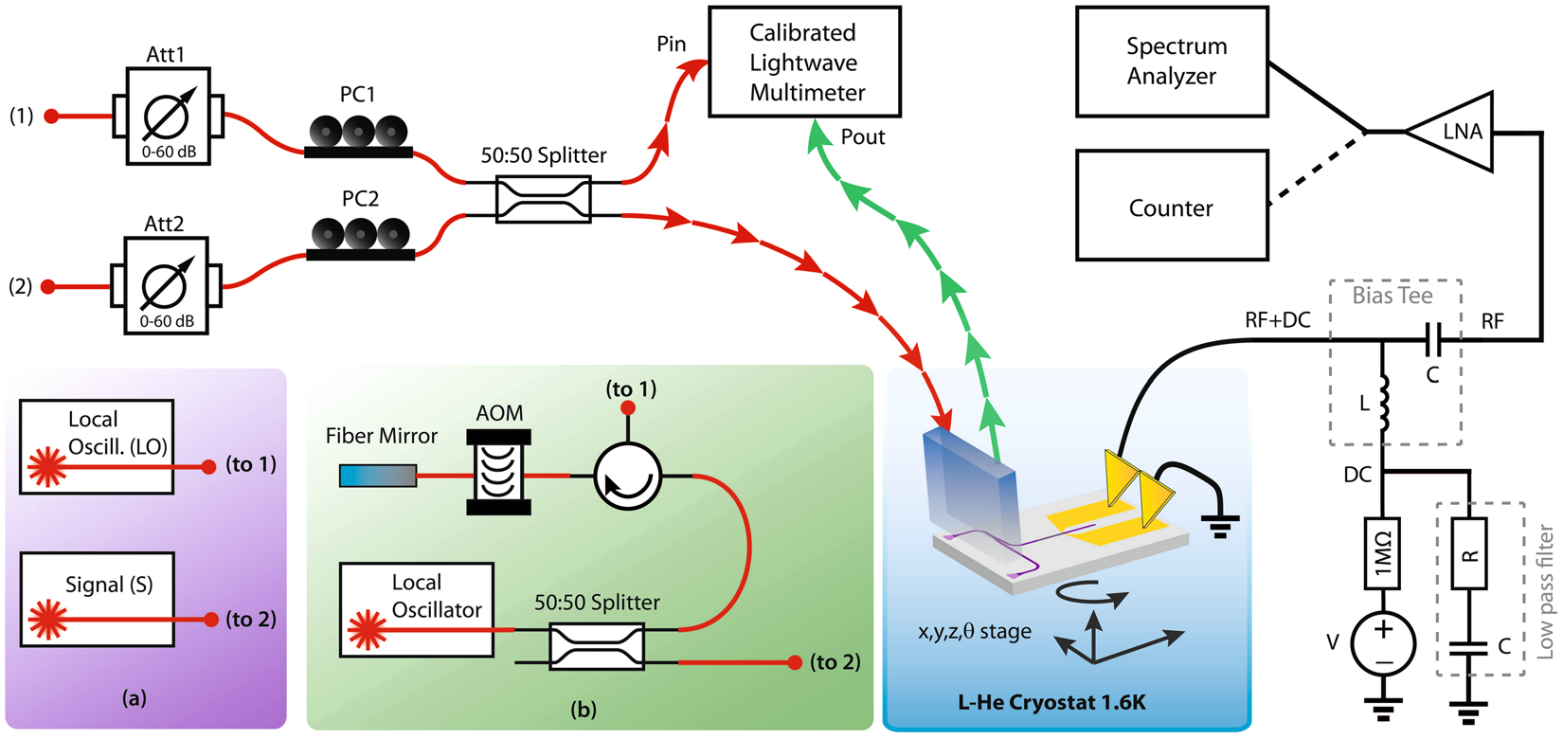
Experimental setups. Schematic view of the experimental setup for the single photon counting and the heterodyne mixing measurement. The nanophotonic device is mounted inside a liquid helium cryostat at a temperature of 1.6 K with optical access (red and green lines and arrows) and electrical access (black lines). (**a**) Coherent detection configuration in which two lasers are connected to points 1 and 2 in the basic scheme. (**b**) Single laser configuration for coherent detection, which allows us to use one laser source as LO and the signal. The generated laser light is divided in two by a 50:50 splitter. One branch of the splitter is directly routed to the mixing setup, while the second one is shifted by ≈400 MHz by the mean of an AOM.

The experimental setup for the characterization of the waveguide integrated single-photon counter is depicted schematically in Fig. 2. A calibrated photon flux is generated from a tunable laser source and attenuated by a calibrated optical attenuator Att_1_ (see Supplementary Materials, S1). A polarization controller PC_1_ is used to adjust the polarization of the fiber mode which is coupled onto the input FGC. The injected power *P*_*in*_ is monitored by a 50:50 beam splitter and an optical power meter. With the same instrument, the output power *P*_*out*_ from the calibration coupler has also been monitored. The measurements are performed according to a standard procedure^8^, which main feature is the ability to determine the photon flux reaching the detector (see Methods for details).

**Figure 3:**
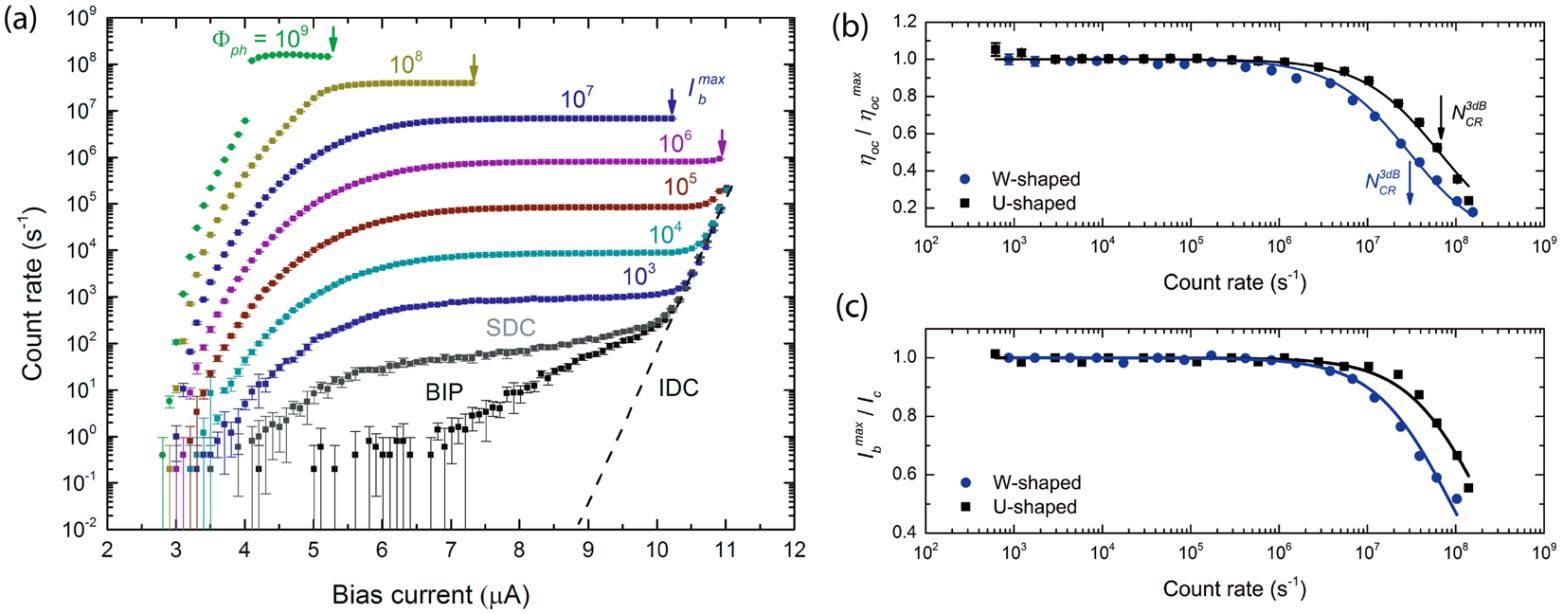
Measurement of the single-photon detector (SNSPD) performance. (**а**) Count rate of the W-shaped nanowire vs bias current, at different levels of optical power at 1550 nm wavelength. The photon flux is shown above each curve and marked by individual colors. The count rate when the laser is turned off but connected to an optical fiber at a room temperature is marked by SDC. The count rate with a closed metal cup is marked as BIP. The intrinsic dark counts marked as IDC (**b**) Dependence of the normalized on-chip detection efficiency (*η*_*oc*_) on count rate. The black squares correspond to the measured data for U-shaped nanowire, while the blue dots for W-shaped nanowire. The fitted values are represented with solid lines and the arrows indicate the count rate ${N}_{CR}^{3dB}$ at which ${\eta }_{oc}({N}_{CR}^{3dB})={\eta }_{oc}^{\max }/2.$ (**с**) Normalized maximum current vs count rate. The black squares represent the measured dependence for U-shaped detector, while the blue dots for the W-shaped detector. The fitted values are represented with solid lines.

The absorption of single photons by the superconducting NbN nanowire leads to a temporal breakdown of the superconducting state and generation of electrical voltage pulses^3, 4^, which, after amplification, are registered by a counter. In Fig. 3a we show the dependence of the count rate (CR) of the WSN detector at different values of Φ_*ph*_, as well as the corresponding dark count rate on the bias current *I*_*b*_. For all curves the CR shows a pronounced plateau. This is indicative of high quality nanowires and implies that the internal detection efficiency saturates. We achieved a maximum efficiency value of *η*_*oc*_ = 86 ± 8% for the WSN and 68% ± 7% for USN setting an input photon flux at the detector of Φ_*ph*_ *≤* 10^6^. We attribute the difference in the detection efficiency for the two detectors to an imporved absorption efficiency for the W-shaped geometry detector. For higher values of Φ_*ph*_ be observe a significant reduction of the *η*_*oc*_ (Fig. 3b). With increasing the photon flux reaching the nanowire, the number of photons impinging the detector during its dead time rises and hence the counting rate significantly decreases. The ${N}_{CR}^{3dB}$ value at which the efficiency drops twice is indicated by the arrows in Fig. 3c and corresponds to a value of 3 × 10^7^ and 6.8 × 10^7^ counts per second for the WSN and USN, respectively. This different behavior can be attributed to the different value of the kinetic inductance *L*_*k*_ of the detectors, which is, by definition, directly proportional to the length of nanowire and inversely proportional to its width^25^.

The same holds for the maximum value of the bias current *I*_*b*_^*max*^ (Fig. 3c). In order to explain this behavior, which has been previously observed by Kerman *et al*.^26^, we have to analyze the properties of the bias circuit in detail. A large number of clicks leads to charging of the capacitor in the bias tee (Fig. 2), introducing an additional bias voltage, in parallel to the main one. The average voltage value depends on the number of counts, on the shape and amplitude of the pulse from SNSPD. Extra current added to the current source leads to an increase of the supercurrent, which translate to a premature suppression of the superconductivity. At low count rates, the influence of the additionally charged capacitor is small, but becomes sufficiently large at count rates greater than 10^7^ ph/s. This leads to the appearance of artifacts in dependence of count rate versus the bias current (see Supplementary Materials, S2), as well as to the need to reduce the bias current of the detector at high CR, and simultaneously reducing the detection efficiency in turn. This relationship allows us to select the optimal bias current conditions for coherent detection.

In addition to the intrinsic dark counts of the detector (IDC) (Fig. 3a), an additional noise contribution results from background induced photons (BIP), generated in the sample chamber from the 300 K parts of the cryostat insert, as well as by hot parts of the optical fiber^27^. The measurement of BIP has been performed with the fiber input closed by a metal cup. The dark count rate of the whole system, indicated as system dark counts SDC, has been performed without sealing the optical fiber input at the room temperature stage, which leads to an enhanced probability of detecting stray light. Improving the optical shielding of the detectors, as well as using a filter system^27^ can significantly reduce SDC in the future down to IDC.

The timing characteristics of the detectors in use, i.e. the nanoseconds decay time (*τ*_*D*_) and picoseconds jitter (*τ*_*J*_) for the WSN, are determined as *τ*_*D*_ = 10 ± 1 ns and *τ*_*J*_ = 56 ± 3 ps and *τ*_*D*_ = 3 ns ± 0.3 ns and *τ*_*J*_ = 60 ± 3 ps for the USN, respectively. The higher decay time for the WSN detectors can be again attributed to the higher kinetic inductance^25^.

Despite the fact that the demonstrated on-chip detection efficiency is quite high (up to 86%), the system detection efficiency (SDE) is limited by the efficiency of the grating coupler *η*_*SDE*_ = 0.86 × 0.15 = 0.13. For applications where higher SDE is required, the design of the FGC can be optimized^28^ or replaced with alternative designs^29^.

### Coherent heterodyne detection with a single laser source

To investigate the SNSPD as a coherent detector the emitted light from tunable laser source is divided into two parts using a 50:50 fiber beam splitter (Fig. 2). The first part acts as the LO, while the second part, routed twice through an acousto-optic modulator (AOM) to relatively shift its frequency to the initial carrier frequency of a constant value of *f*_*IF*_ ≈ 400 MHz, acts as a signal S (Fig. 2b). Both light inputs from the LO and S are attenuated (Att_1_, Att_2_), routed through a polarization controller (PC_1_, PC_2_) and combined at the fiber optics beam splitter. A fiber array is used to conduct the light to the nanophotonic devices, placed on a motorized stage (AttoCube Systems) in a cryostat at 1.6 K temperature (see Methods for details).

To analyze the intermediate frequency signal we used a spectrum analyzer (Rohde & Schwarz ZVL6). This method allows us to directly visualize the spectrum of the SNSPD detection pulses and evaluate the IF signal as well as all the contributions from the different noise sources. The voltage trace (pulses from SNSPD) is Fourier-transformed by the spectrum analyzer, and the square of the modulus of the Fourier-transform is represented as power (see Supplementary materials, S3).

The IF spectrum, obtained as a Fourier transform of the pulses train by the SA, includes (1) electrical noise of the system, (2) the power spectrum of a single pulse proportional to the total incident power (*P*_*LO*_ + *P*_*S*_), and (3) the coherent signal due to the LO and S field beating, at which detection, we are interested on.

**Figure 4:**
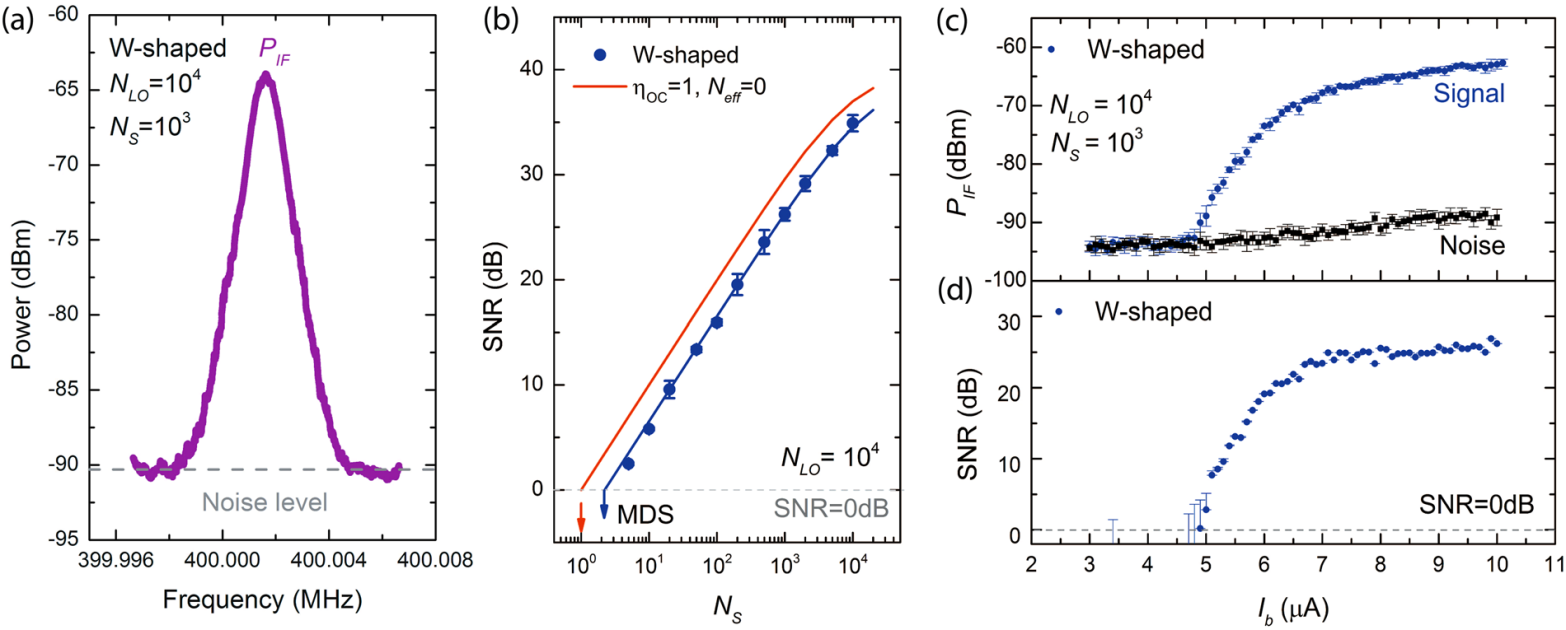
Measurement of the SNSPD performance as coherent detector. (**a**) Measured power vs frequency in the frequency range of *f*_*IF*_ ± 5 kHz. (**b**) Measured SNR vs different signal *N*_*S*_ at fixed *N*_*LO*_ = 10^4^ (blue dots). The red line indicates the simulated curve for an ideal shot-noise limited photodetector. The arrows indicate the *N*_*S*_ when SNR = 0 dB. (**с**) IF power for signal and noise vs bias current. (**d**) Extrapolated SNR vs bias current.

In a first step of the measurement protocol we select the desired resolution bandwidth (RBW) of the SA. By varying the RBW and measuring the IF power at *f*_*IF*_ = 400.016 MHz, we observed that the IF power does not significantly depend on the RBW down to 1 kHz. This indicates that the signal width at the IF is narrower. The signal amplitude is not cut out significantly for RBW ≥ 1 kHz (see Supplementary Materials, S4) and allows us to achieve high spectral resolution equal to *f*/*∆f* ≈ 193.5 THz/1 kHz ≈ 1.935 × 10^11^. The IF power of the WSN output for a RBW = 1 kHz is depicted in Fig. 4a. The spectrum shows a clear signal *P*_*IF*_ at the beat frequency *f*_*IF*_ = 400.016 MHz as well as the noise level (*P*_*noise*_) (gray dashed line). The noise contribution mainly arises from two sources: the amplifier noise, and the power noise pulses generated by the detector (SDC and counts from LO, S).

The signal-to-noise ratio for the IF power detected by the SA can be defined as (see Supplementary Materials, S3):

1 $$SNR={\eta }_{OC}{m}^{2}\frac{{N}_{S}{N}_{LO}}{{N}_{S}+{N}_{LO}+{N}_{SDC}+{N}_{eff}}\equiv {\eta }_{OC}SN{R}_{1},$$

where *m* ^2^ is the mixing efficiency^19, 20^. This value indicates how well the electromagnetic fields of the LO and the S overlap in the single-mode waveguide with the detector placed atop of it. For our analysis we assume *m* ^*2*^ = 1. *N*_*S*_, *N*_*LO*_, *N*_*SDC*_, *N*_*eff*_ are the average numbers of photons collected in the time interval *t* = 1/RBW respectively from S, LO, system dark counts (including intrinsic dark counts, room stray light as well as background induced photons) and the effective electronics noise at IF. The total integration time in the frequency range of *f*_*IF*_ ± 5 kHz, including 10 points with accumulation time *t* = 1/RBW is equal to *t*_*total*_ = 10 × 1 ms = 10 ms. This mode can be used in applications when a quick response is needed. In our case, when a greater accuracy were preferred at the expense of time, the total integration time was 20 sec (Fig. 4a). The additional internal approximation by 32001 points, performed by the SA, meanwhile, does not affect the total accumulation time.

Experimentally the SNR can be determined as:

2 $$SNR=({P}_{IF}-{P}_{noise})/{P}_{noise},$$

where *P*_*IF*_ and *P*_*noise*_ are the power of IF signal and noise respectively, measured by SA. In order to find the best operating condition, we first measured the dependence of *P*_*IF*_ versus *I*_*b*_ at fixed value of Φ_*LO*_ = 10^7^ ph/s. This photon flux corresponds to an average number of photons per integration time *N*_*LO*_ = Φ_*LO*_/RBW = 10^4^ (Fig. 4c). In Fig. 4d we show the measured dependence of SNR vs *I*_*b*_. The SNR increases with increasing *I*_*b*_ and shows the same plateau behavior as the on-chip detection efficiency of the detector, presented in Fig. 3a. The two quantities are indeed correlated: at higher bias currents, the detection efficiency increases and consequently also the power at the intermediate frequency is also increased.

Keeping *I*_*b*_ constant at the corresponding maximum value of *P*_*IF*_ and setting a constant photon flux of the local oscillator *N*_*LO*_, we determined the dependence of the SNR on the *N*_*S*_. The experimental results and the fit to equation 1 are shown in Fig. 4b. When *N*_*S*_, *N*_*SDC*_, *N*_*eff*_ are small enough such that *N*_*LO*_ *» N*_*S*_ + *N*_*SDC*_ + *N*_*eff*_, equation (1) reduces to SNR = *η*_*OC*_ *N*_*S*_. The SNR depends linearly on *N*_*S*_ and starts to saturate when *N*_*S*_ ≈ *N*_*LO*_. If *N*_*S*_ = *N*_*LO*_ and *N*_*SDC*_, *N*_*eff*_ ≈ 0, then is reduced by half: SNR = *η*_*OC*_ *N*_*S*_/2. The same graph shows the calculated dependence of the SNR(*Ns*) for an ideal shot-noise limited mixer with *η*_*OC*_ = 1, *m* ^*2*^ = 1, *N*_*SDC*_ = 0, *N*_*eff*_ = 0. The arrows indicate the minimum detectable signal (MDS) for both cases, when the SNR = 0 dB. This corresponds to the condition *P*_*IF*_ = 2 × *P*_*noise*_, for the equation 2.

**Figure 5:**
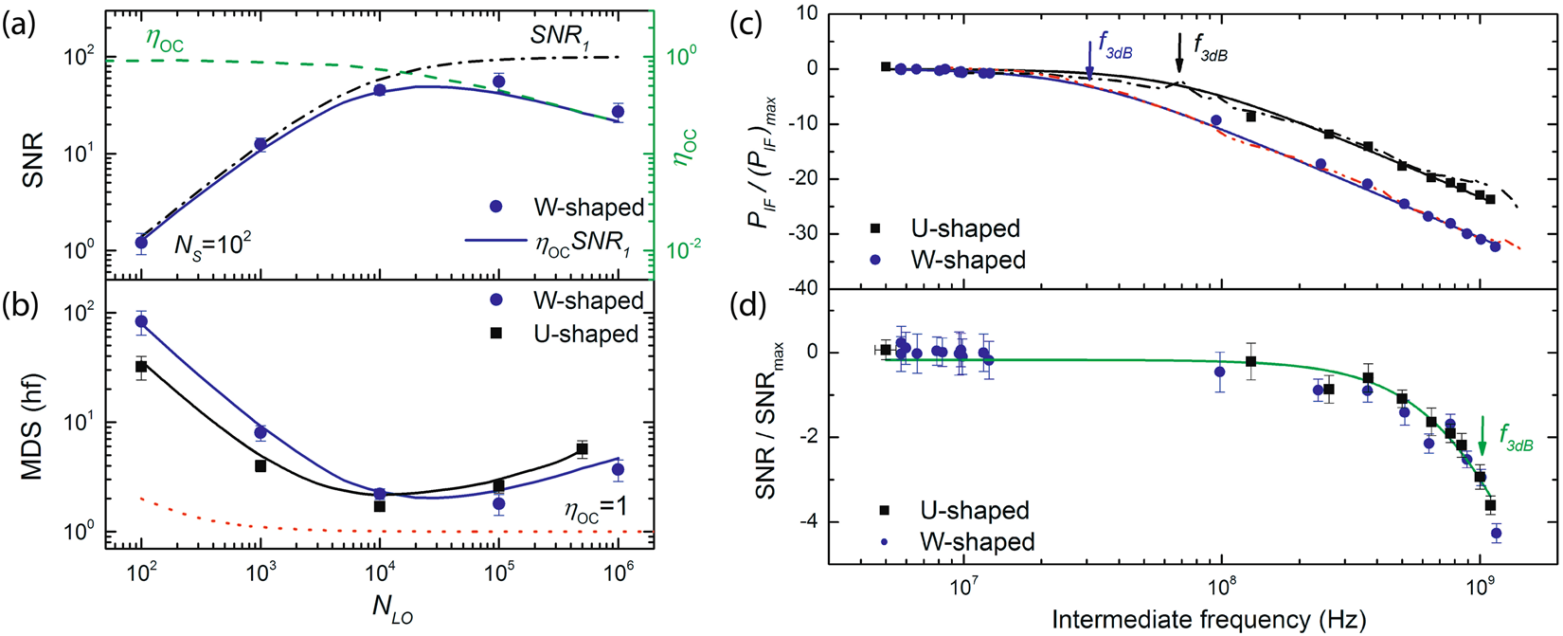
Measurement of the SNSPD performance as coherent detector. Blue dots correspond to the W-shaped nanowire and black squares to U-shaped nanowire. (**a**) Measured SNR vs *N*_*LO*_ at a constant signal *N*_*S*_ = 10^2^. The green dashed line shows the measured dependence of *η*_*oc*_ (*N*_*LO*_) and the black dash-dotted line represents the calculated SNR_1_ with extracted data of *N*_*eff*_. The blue solid line is the result of multiplication of *η*_*oc*_ (*N*_*LO*_) and SNR_1_ (**b**) Minimum detectable signal (MDS) in terms of single photons vs *N*_*LO*_. The red dotted line shows the MDS for ideal shot noise limited detector (**c**) Measured IF bandwidth for both types of detectors. The dash-dotted lines show the Fast Fourier transform of a pulse detector. The fitted values are represented with solid lines and the arrows indicate the IF frequency *f*_*3dB*_ at which power decreases in two times. (**d**) Noise bandwidth vs *f*_*IF*_. The fitted values are represented with the green solid line and the arrows indicate the IF frequency *f*_*3dB*_ at which SNR decreases in two times.

The same procedure has been applied for different *N*_*LO*_. Figure 5a shows the dependence of the SNR(*N*_*LO*_) at a fixed *N*_*S*_ = 10^2^ for W-shaped detector. For small values of *N*_*LO*_ the relationship is linear, while it saturates and even decreases at larger values. The growth of the SNR with increasing *N*_*LO*_ can be associated with the overcoming of the effective noise of the electronics *N*_*eff*_, while the drop can be associated with a reducing detection efficiency with increasing photon flux. According to the experimentally measured dependence of the PSD vs an incident photon flux, we have estimated the value of the effective noise *N*_*eff*_ for both types of nanowires at measured IF frequency 400.016 MHz (Supplementary Materials, S5). We found *N*_*eff*_ = 7 × 10^3^ for W-shaped and *N*_*eff*_ = 2.6 × 10^4^ for U-shaped detectors at the same RBW = 1 kHz.

The detectable signal reaches its minimum at values *N*_*LO*_ ≈ 10^4^–10^5^ for both types of the detectors. The minima of the MDS is about 1.8–2.3 times the photon energy and close to MDS = 1 for the shot-noise limited mixer (Fig. 5b).

### Coherent heterodyne detection with two laser source and IF bandwidth measurement

The same experimental setup which we used for characterizing the device sensitivity can be re-adapted for heterodyne detection using two lasers sources instead of one^18^ (Fig. 2a). We employ two different lasers, one for generating the S signal at 1550.000 nm and a second for the LO signal at 1550.001 nm, corresponding to an initial frequency shift of ≈125 MHz. The photon flux was kept constant at Φ_S_ = 10^7^ ph/s and Φ_LO_ = 10^8^ ph/s. As previously asserted, mixing the signal of two different laser sources limits the IF frequency stability and therefore the measurements were carried out at RBW = 1 MHz in this case.

The measured *P*_*IF*_ versus *f*_*IF*_ is shown in Fig. 5c. The upper frequency limit of the conversion bandwidth of the mixer is defined as the cut-off frequency (*f*_*3dB*_), corresponding to a decrease in IF signal power *P*_*IF*_(*f*_*IF*_ = 0)/*P*_*IF*_(*f*_3*dB*_) = 2 and has been derived by fitting the intermediate frequency power spectra using the formula *P*_*IF*_ = *P*_*IF*_(*f*_*IF*_ = 0) − 10 log_10_(1 + (*f*_*IF*_/*f*_3*dB*_)^2^). The obtained conversion bandwidth for W-shaped detector was 29 ± 3 MHz, and for the U-shaped detector 68 ± 7 MHz. These values correspond to the ${N}_{CR}^{3dB}$ at which the detector efficiency drops by a factor of two (Fig. 5c).

In addition to the conversion bandwidth we determined the noise bandwidth, defined as the intermediate frequency value at which the SNR is reduced by 3 dB, shown in Fig. 5d. With respect to the conversion bandwidth, the noise bandwidth does not depend on the detector length and reaches an upper frequency limit of 1040 ± 95 MHz. We associate the measured data with the amplifier bandwidth, while the theoretical prediction^21^ shows a jitter limited noise bandwidth equal to about ${f}_{jitter}^{3dB}=2\,\mathrm{ln}\,2/{\tau }_{J}\approx 7.35$ GHz for the detectors. This prediction is encouraging for coherent detection with IF bandwidth for various applications, with the use of wide-bandwidth low noise cold amplifiers in the future.

## Discussion

The calculated dependence of the minimum detectable signal on the local oscillator power *N*_*LO*_ for an ideal shot noise limited photodetector (red dash-dotted line), with unity detection efficiency as well as in complete absence of noise, is reported in Fig. 5b. When the LO power is much higher than the signal (*N*_*LO*_≫ *N*_*S*_), the incident signal integrated over the resolution bandwidth corresponding to the MDS equals *hf* and is independent of the LO power. This value increases to 2 × *hf* for *N*_*LO*_ = *N*_*S*_. However, we observed a dependence that differs from the ideal one (Fig. 5b). This can be explained considering the detector recovery time and noise contribution. At high photon flux, the detector does not have time to recover the original superconducting state, and *η*_*OC*_ is significantly reduced. This leads to a decrease of the SNR and an increase of the MDS, respectively. A reduction of the *η*_*OC*_ determines a practical upper limit to the LO power which can be used at the highest detection performance. Increasing the speed of the detector enables not only to increase the dynamic range for the LO power, but also to increase the conversion bandwidth (Fig. 5c). This can be done by reducing the length of the detector without compromising absorption and the IDE by embedding the detector inside a cavity^30^, as well as through the optimization of the geometric parameters of the waveguide. On the other side, the LO power lower limit depends on the presence of additional noise in the system. Since the dark counts of the SNSPD are very small (for example, at *I*_*b*_ < 10 µA, SDC/RBW < 1, see Fig. 2), the main noise contribution is the noise introduced by the experimental setup, especially the amplifiers at high frequencies (*N*_*eff*_), which can be estimated by the power spectral density (Supplementary Materials, S5). Knowing *N*_*eff*_, the SNR_1_ can be then extracted (see equation 1) and by multiplying SNR_1_ (Φ_LO_) and *η*_*OC*_ (Φ_LO_) we found a good agreement with the observed dependence of the SNR (*N*_*LO*_) and MDS (*N*_*LO*_).

Concerning the improvement of the detection procedure, we showed that the spectrum of the SNSPD pulse adds a noise contribution *P*_*noise*_ to the *P*_*IF*_. The output power from the photon-counter includes the detector pulse itself (see Supplementary Materials, S3). Hence, by replacing an exponentially decaying pulse with a delta-function pulse, one could diminish the output noise related to the pulse spectrum. This can be done by registering the detection pulse arrival time points with a counter and performing the Fourier transform of this signal. Although this approach will not lead to an increase in the detector speed, which is determined mainly by the kinetic inductance^25^, the IF bandwidth will not repeat the exponential pulse spectrum (Fig. 5c) and will be determined by the system noise, in analogy to the SNR bandwidth (Fig. 5d). Wide IF bandwidth is especially useful when operating devices without calibration, as well as for minimizing the influence of the *N*_*eff*_ at high frequencies (Supplementary Materials, S5).

Also, an advantageous spectrum analysis of wide-band signals can be carried out by combinational use of integrated-optics based wavelength dividers^12–17^ allowing for partitioning the signal into discriminated spectrum-channels and fine spectrum analysis by the proposed heterodyne technique set up at each of the channels.

The coherent detection method demonstrated in this paper could also be successfully accomplished with SNSPDs implemented on a variety of material platforms^9^ as well as other types of single-photon detectors, such as the transition edge sensors (TES)^31, 32^ or commercially available free-running APDs^33, 34^. As a matter of fact, successful integration of a TES detector on an optical waveguide has already been demonstrated. Compared to SNSPDs, TES operation requires sub-kelvin temperatures with increased equipment costs. Concerning free-running APDs, considerable dark count rates (up to 6 kHz)^33^ and afterpulsing probability (>5%)^34^ limit the achievable MDS. In addition, integration on chip has not been demonstrated yet with APDs. Significant dead time (at a level of 0.1–100 μs) for TES and APDs leads to increased signal acquisition times, while lower timing resolution (>100 ps) decreases the achievable ${f}_{jitter}^{3dB}$ bandwidth. In contrast, SNSPDs provide high on-chip detection efficiency, a low dark count rate, and high temporal resolution in a single device which makes them attractive for coherent detection on chip.

In conclusion, we have demonstrated heterodyne mixing with single-photon counting detector embedded in a travelling wave geometry approach, where the NbN nanowire is evanescently coupled to a waveguided mode field. We demonstrated single-photon counting with *η*_*oc*_ up to 86% and a coherent mixing with a spectral resolution (*f*/*∆f*) greater than 10^11^ at telecom wavelengths, operated close to the shot-noise limit. We further experimentally showed heterodyne detection at ultra-low local oscillator power (10^5^–10^9^ ph/s) and an extremely weak signal photon flux of the incident test signal (4 × 10^3^–10^9^ ph/s). A clear dependence between the conversion bandwidth and the nanowire geometry has been observed. In particular, shorter wires (U-shaped) showed greater throughput than longer (W-shaped) and can be used for coherent detection applications where narrow-line observations are required in a wide band. Lastly we commented on possible ways to further improve the performance of these detectors, that can be adopted for coherent detection in both classical and quantum optics.

## Methods

### Device fabrication

The integrated hybrid architecture including waveguides and superconducting nanowire detectors (SNSPDs) is realized on commercially available silicon wafers Si (350–400 µm) with a thermal silicon oxide of SiO_2_ (2600 nm) and silicon nitride Si_3_N_4_ (450 nm) grown on top. After cleaning the surface of the substrate we deposit an ultra-thin niobium nitride (NbN) film with a nominal thickness of 4 nm ± 0.5 nm. The deposition is made by a reactive magnetron sputtering in Argon and Nitrogen atmosphere. We reached a maximum critical temperature *T*_*c*_ = 9.5 K for films deposited at a substrate temperature *T*_*S*_ = 800 °C with partial pressures of Argon and Nitrogen of 6 × 10^−3^ and 2.5 × 10^−4^ mbar, respectively. The sheet resistance of the deposited NbN film measured at room temperature is 620 Ohms/sq. After the superconducting NbN film deposition, nanophotonic devices are fabricated using three e-beam lithography steps. In the first step contact pads and alignment marks in a positive e-beam polymethyl methacrylate (PMMA) resist are defined. Then, by e-beam physical vapor deposition (ePVD) we deposit 5 nm of chromium (Cr) as an adhesion layer and 150 nm of gold (Au) and finalize the contact pads and the alignment marks by lift-off in acetone. In the second step, NbN nanowires are realized using HSQ resist and CF_4_ reactive ion etching. The final step consists in patterning rib-waveguides into ma-N 2403 resist by e-beam lithography and obtaining the final device by dry etching the Si_3_N_4_ layer by RIE in CHF_3_ plasma. Residual ma-N 2403 resist is then removed by an additional O_2_ plasma cleaning step. In our case, the variation in thickness of the NbN superconductor film, as well as the quality of electron beam lithography affect the device yield the most. For this reason, we usually fabricate arrays of devices with slightly different parameters. Over a 2 × 2 cm sample we obtain device yield at a level of about 70%.

### Measurement setup (single photon counting)

To generate a calibrated photon flux we used a tunable laser source (New Focus TLB 6600) attenuated by an optical attenuator Att_1_ (HP 8156A). A polarization controller PC_1_ (Thorlabs FPC032) is used to adjust the polarization of the fiber mode which is coupled onto the input FGC. The injected power *P*_*in*_ is monitored by a 50:50 beam splitter and a calibrated Lightwave multimeter (HP 8163A). With the same instrument, the output power *P*_*out*_ from the calibration coupler has also been monitored. The photon flux reaching the detector can be written as Φ_det_ = (*P*_*in*_/*hf*) × *S* × *C* × *WT*_2_. Where *S* is the splitting ratio of the on-chip 50:50 Y-splitter, *C* is the coupling efficiency, *WT*_*2*_ waveguide transmission of the arm leading to the nanowire. The coupling efficiency is determined from the input and output powers as: $C=\sqrt{{P}_{out}/({P}_{in}\times S\times W{T}_{1})}$, where *WT*_*1*_ refers to the waveguide transmission for the calibration arm. The electrical readout circuit is composed of a stable current source CS (Keithley 2400), Mini-Circuits Bias Tees (ZFBT-GW6+) to separate RF and DC components, an RC filter and two low noise amplifiers (Mini-Circuits ZFL-1000LN+). The absorption of single photons by the superconducting NbN nanowire leads to a temporal breakdown of the superconducting state and generation of electrical voltage pulses, which, after amplification, are registered by a 225 MHz counter (HP 53132A). The on-chip detection efficiency is then determined as *η*_*oc*_ = (*CR* − *SDE*)/Φ_det_, where CR is the count rate and SDC is the system dark count rate.

### Measurement setup (heterodyne detection)

For demonstration of the on-chip coherent detection we use tunable laser source (TLS Santec 510). The emitted light is divided into two parts using a 50:50 fiber beam splitter. The first part acts as the LO, while the second part, routed twice through an acousto-optic modulator (AOM Gooch&Housego fibre Q) to relatively shift its frequency to the initial carrier frequency of a constant value of *f*_*IF*_ = 400.016 MHz, acts as a signal S (Fig. 2b). Both light inputs from the LO and S are attenuated (Att_1_, Att2), routed through a polarization controller (PC_1_, PC_2_) and combined at the fiber optics beam splitter. A fiber array is used to conduct the light to the nanophotonic devices, placed on a motorized stage (AttoCube Systems) in a cryostat at 1.6 K temperature. The calibration of the radiation power incident on the input of the nanophotonic devices *P*_*in*_ is performed separately for each channel by switching the attenuators Att_1_ and Att_2_ off separately and by measuring the output power under the two respective input conditions. We note that the same characterization could also have been performed with two different lasers, one as source and a second one as local oscillator. We opted, however, for a single source in order to minimize the contribution of the frequency fluctuation of the laser output that could limit the detection resolution bandwidth and the overall system sensitivity at *f*_*IF*_. For the heterodyne detection with two laser source and IF bandwidth measurement, we replaced in the main scheme the AOM and mirror with a second tunable laser source (New Focus NF6427).

## Electronic supplementary material

Supplementary Information
