# Supplementary material for: On-chip coherent detection with quantum limited sensitivity
Source: Sci Rep. 2017 Jul 6;7:4812. doi: 10.1038/s41598-017-05142-1 (PMC5500578; doi:10.1038/s41598-017-05142-1)
Supplement: Supplementary file 1 — Supplementary Information [file 41598_2017_5142_MOESM1_ESM.doc]

**Supplementary Materials**

**List of contents**

**S1. Calibration of the optical attenuators**

**S2. Modeling of the measured artifacts at high count rates**

**S3. Conversion of the signal by the spectrum analyzer and calculation of the SNR**

**S4. Stability of the intermediate frequency**

**S5. Estimation of the effective electrical noise**

**References**

**S1. Calibration of the optical attenuators**

In all the presented experiments, we used two tunable attenuators (HP 8156A) in order to set the incident photon flux at the nanowire position. For their calibration we used a tunable laser source (New Focus TLB 6600) and a power meter (HP 8153A). We measured the transmitted power from the laser to the power meter for different attenuation values. The input power of the laser was set at the level of 0 dBm @ 1550 nm. Both attenuator have been calibrated separately over the entire operating range (0 to 60 dB). The measured data are shown in Figure S1a,b. To check the possible interference between the two attenuators, we also characterized their series configuration, keeping one of the two attenuators fixed (Figure S1c). In the range from 0 to 60dB the attenuators do not affect each other, and the slope of the calibration line is close to the slope of the individual attenuators. We could not test the linearity for attenuation greater than 60 dB because we were limited by the sensitivity of the power meter.


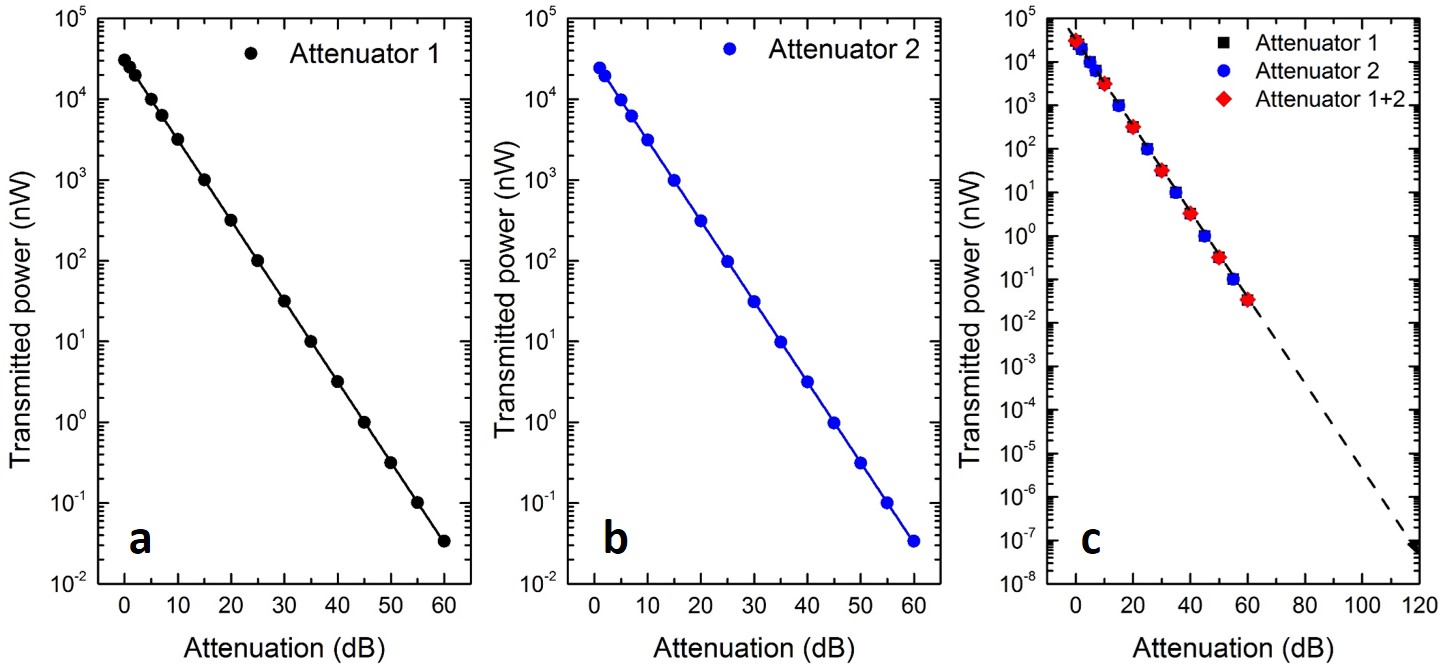


**Supplementary Figure S1.** Calibration of the optical attenuators. For all the graphs points (circles, squares, rhombs) are the measured data, line is the linear fit. **(a)** Attenuator 1. **(b)** Attenuator 2. **(c)** Attenuator 1, Attenuator 2 and Attenuator 1+2.

**S2. Modeling of the measured artifacts at high count rates**

We observed some artifacts in the count rate vs bias current curve at high photon flux (Figure 2 main text). In order to explain this behavior, we have to analyze the properties of the adopted bias circuit. A block diagram of the electrical connection of the SNSPD is shown in Figure S2a. The bias current is produced by a voltage source (Keythley2400) with 1MOhm resistor in series. A bias tee (MiniCircuits ZFBT-GW6+) is used for splitting the DC bias and the RF signal. The RF signal coming from the detector is amplified (MiniCircuits ZFL-1000LN+) and supplied to the counter, the SA or to the oscilloscope. Key elements which influence the count rate are the capacitor in the bias tee as well as amplifier input capacitor.

Since the capacitors filter any DC component, the output voltage sent to the amplifiers, for a long integration, is always zero. The average voltage value depends on the number of counts, and on the shape and amplitude of the pulse from SNSPD. In first order approximation, this voltage can be considered directly proportional to the number of pulses from the detector. A large number of clicks leads to the fact that the capacitor is charged, introducing and an additional bias source in parallel to the main one. At low count rates the influence of a charged capacitor is small, but becomes sufficiently large at count rates greater than 10^7^ ph/s. This leads to the appearance of artifacts in dependence of count rate versus the bias current as well as to a loss of the superconducting state before the *I_C_*, observed at low CR. Mathematically, this mechanism can be described by the expression:

$CR=\Phi_{ph}\times\eta_{OC}^{*}(I_{b})=\Phi_{ph}\times\eta_{OC}(I_{b}+CR(I_{b}+I_{add})\times k)$ (SE1)

where *k* is an adjustable parameter that includes the amplitude of the output pulse, the gain, pulse shape and the resistance of the detector in the momentum of a click. And *I_add_* describes the additional bias current flowing because of the charged capacitor. The formula above is iterative and requires a consistent calculation of $\eta_{OC}^{*}$.


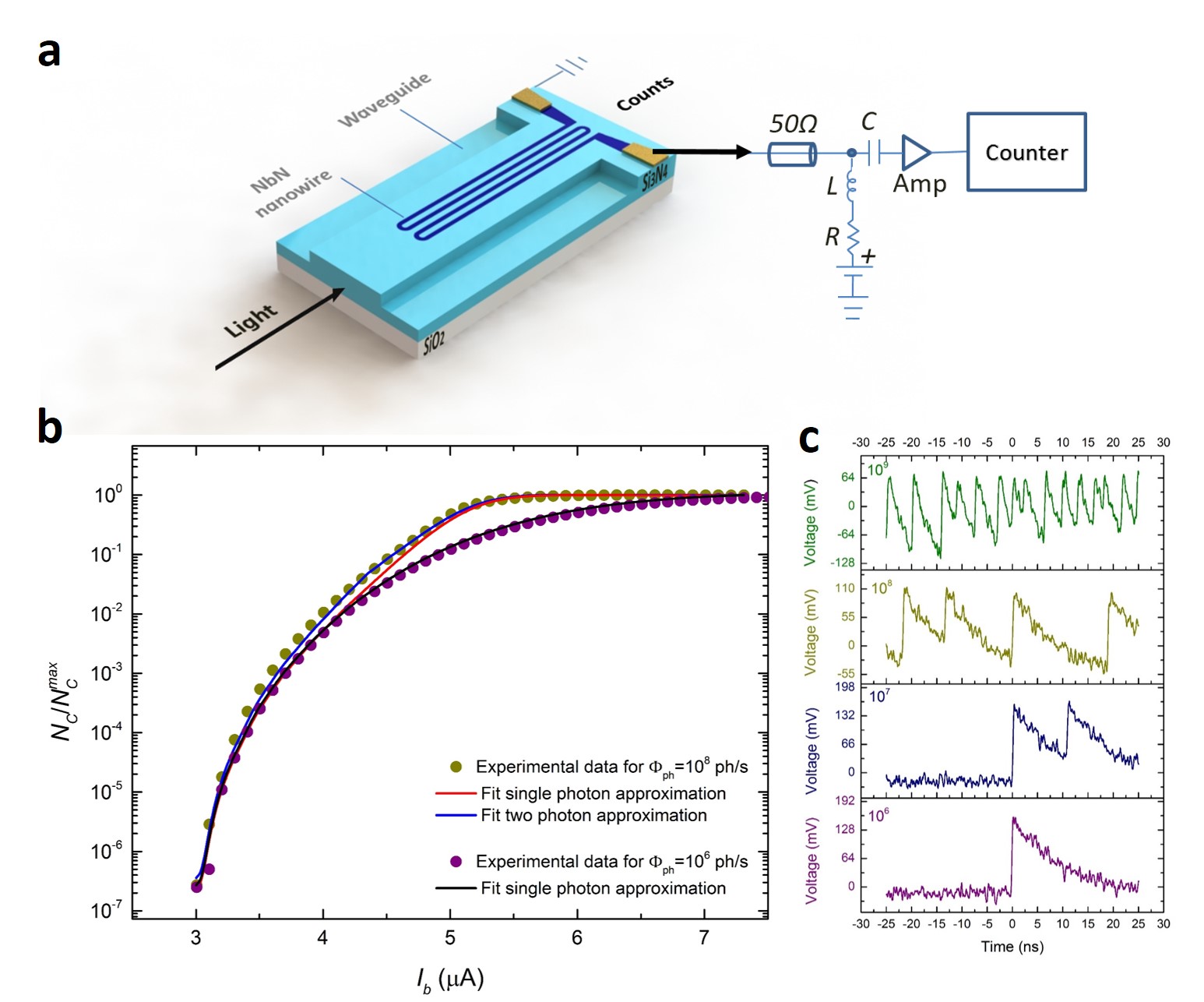


**Supplementary Figure S2. (a)** Schematic view of the electrical readout circuit connected to W-shaped nanowire. **(a)** Normalized count rate vs bias current for an input photon flux of 10^8^ ph/s (dark yellow dots) and for 10^6^ ph/s (purple dots). The red and black curve represent the single photon approximation fit for 10^8^ ph/s and 10^6^ ph/s respectively, while the blue curve represents the fit considering also the multiphoton detection contribution. **(c)** Detection pulses train for different input powers. The more the pulses rate increases, the more a drop in the electrical signal towards negative values is visible. This is indicative of a change in the bias conditions for high optical input power.

Figure S2b shows the normalized experimental data of the CR (from Figure 3, main text) vs the bias current (*I_b_*) for two photon flux equal to 10^8^ ph/s (dark yellow dots) and of 10^6^ ph/s (purple dots). The black curve in Figure S2b corresponds to the fit to equation S1 and is a good description of the experimental data for 10^6^ ph/s. The red curve describes the behavior of the CR at 10^8^ ph/s well at high currents. However, at low bias currents (< 5 µA), the discrepancy with the experimental data is sufficiently large. We associate this with the transition to a 2 photons detection regime^2^. It is then possible to better fit the experimental data considering also the 2 photon detection regime contribution, as depicted in the blue curve in Figure S2b.

**S3. Conversion of the signal by the spectrum analyzer and calculation of the SNR**

**S3.1. Signal conversion**

The signal received from the detector is a voltage trace *V(t)* containing identical voltage pulses *v(t)* at different moments of time *t_n_* and some noise added by the amplifiers. Let us first omit the noise added by the amplifiers and write

$V\left(t\right)=\sum_{n=1}^{N} v\left(t-t_{n}\right)$. (SE2)

where *N* is the number of counts during the accumulation time *T*. This voltage trace is Fourier-transformed by the spectrum analyzer (SA), and the square of the modulus of the Fourier-transform is represented as power spectral density (PSD). The Fourier-transform of eq. SE2 reads

$V\left(\omega\right)=\sum_{n} e^{-i\omega t_{n}}v\left(\omega\right)$ (SE3)

where *v*(ω) is the Fourier-transform of *v*(*t*), i.e. spectrum of the voltage of a single pulse of the detector.

The time bins *t_n_* are distributed non-deterministically, but the probability that a pulse to occurs at a certain instant *t* is proportional to the power reaching the detector at *t*. In case of two monochromatic light waves, with a reciprocal frequency shift *f_IF_ (*or angular frequency *Ω = 2πf_IF_)*, the power reaching the detector is $P_{S}+P_{LO}+2\sqrt{P_{S}P_{LO}}\cos(\Omega t+\phi)$ and the pulse probability is

$p\left(t\right)=\Phi_{ph}\eta_{OC}\left(1+\alpha\cos(\Omega t+\phi)\right)$. (SE4)

The modulation depth of the beating is: $\alpha=2\sqrt{P_{S}P_{LO}}/\left(P_{S}+P_{LO}\right)=2\sqrt{M_{S}M_{LO}}/\left(M_{S}+M_{LO}\right)$, where *M_S_* and *M_LO_* are the average number of photon counts during the time *T*, generated by the signal and the local oscillator individually.

To calculate the power spectral density given by the spectrum analyzer, one should take the square of the Fourier-transformed signal and average it over all possible values of *t*_1_, *t*_2_, …, *t_n_*, taken with their probabilities *p*(*t_n_*). The result is:

$\left\langle\left|V\left(\omega\right)\right|^{2}\right\rangle=\left|v\left(\omega\right)\right|^{2}\left[M_{S}+M_{LO}+M_{S}M_{LO}\delta_{\omega\Omega}\right]$ (SE5)

with *δ_ωΩ_*=1 for *ω*=*Ω* and 0 elsewhere. The sum (SE3) has nonzero average if only period of the phase of the exponents is correlated with the period of oscillations of *p(t)*, i.e. if *ω*=*Ω*; otherwise, eq. SE3 is a sum of terms with stochastic phases, which average is zero, therefore the average of its square is proportional to the average number of terms in the sum, *M = M_S_ + M_LO_*.

For a data accumulation time *T* longer than the beating coherence time, or equivalently if the spectral resolution (RBW) of the analyzer is small compared to the spectral width of the beating signal *Δω*, eq. SE4 can be generalized to

$\left\langle\left|V\left(\omega\right)\right|^{2}\right\rangle=\left|v\left(\omega\right)\right|^{2}\left[M_{S}+M_{LO}+M_{S}M_{LO}f\left(\omega\right)\right]$ (SE6)

where *f*(*ω*) the power spectrum of the beating.

The spectrum of exponentially decaying SNSPD pulse limits the bandwidth *v(ω)* representing the spectrum of the single pulse in eq. SE6 turns to zero the IF spectrum also turns to zero. In practical applications, exponentially decaying pulses could be replaced by delta function pulses by registering the detection pulse arrival time points with time measuring electronics and performing the Fourier transform of this signal. In this case *v(ω)* in eq. SE6 will not depend on frequency *ω* any more and will not limit conversion bandwidth. For this reason 3dB roll-off in Fig. 5c (in the main text) should move to higher frequency and become ultimately equal to 3dB roll-off of SNR bandwidth (Fig. 5d in the main text).

**S3.2. Signal-to-noise ratio**

In the limit of a narrow-band beating signal, *Δω* < *RBW*, it is natural to define the signal-to-noise ratio as *SNR* = (*PSD*(*ω* = *Ω*) − *PSD*(*ω* ≠ *Ω*))/*PSD*(*ω* ≠ *Ω*). Using eq. SE6, one obtains:

$SNR=\frac{M_{S}M_{LO}}{M_{S}+M_{LO}+M_{SDC}}$, (SE7)

where *M_SDC_* is the system dark counts rate, including the intrinsic dark counts of the detector *M_IDC_* a poor shielding from ambient illumination, as well as background induced photons *M_BIP_*, generated in the sample chamber from the 300K parts of the cryostat insert and by hot parts of the optical fiber^3^ (Figure 3a in the main text).

It is important to notice that the pulse spectrum drops out from equation eq. SE7. Therefore, differently for the signal and the noise bandwidth, the SNR is not limited by the pulse spectrum width. In principle, eq. SE7 predicts infinite noise bandwidth. For a real detector, the quantity that limits the noise bandwidth is the timing jitter *τ_J_*, i.e. the scatter of moment of the click *t* with respect to the moment of the photon absorption. This scatter smears the oscillations probability eq. SE3 in scale of *τ_J_* and leads to a decrease of the signal and the SNR at the frequency *ω*>*2π/τ_J_*. In the case of Gaussian shape jitter the 3dB cut-off frequency $f_{jitter}^{3dB}=2\ln2/\tau_{J}=7.35$ GHz^4^.

**S4. Stability of the intermediate frequency**

We studied power stability at the intermediate frequency in two different ways. First, we investigated the dependence of *P_IF_* vs resolution bandwidth (RBW) of the spectrum analyzer. It allows us to select the minimum RBW in which the signal has not yet been reduced. The better stability of *P_IF_* corresponds to the smaller RBW and provides a possibility to detect weaker photon fluxes. On the other hand, we investigated the dependence of power at an intermediate frequency according to the frequency difference signal and the local oscillator laser. This allowed us to select the tuning range in which it is possible to measure the conversion of band detector without power adjustment in automatic mode.

**S4.1. Intermediate frequency power vs resolution bandwidth**

In Figure S3 we show the IF power at *f_IF_* = 400 MHz measured at the different resolution bandwidth. We observed that the IF power does not significantly depend on the RBW up to 1 kHz. This indicates that the signal width at the IF is narrower and RBW down to 1 kHz can be used for measurement the performance of the coherent detector.


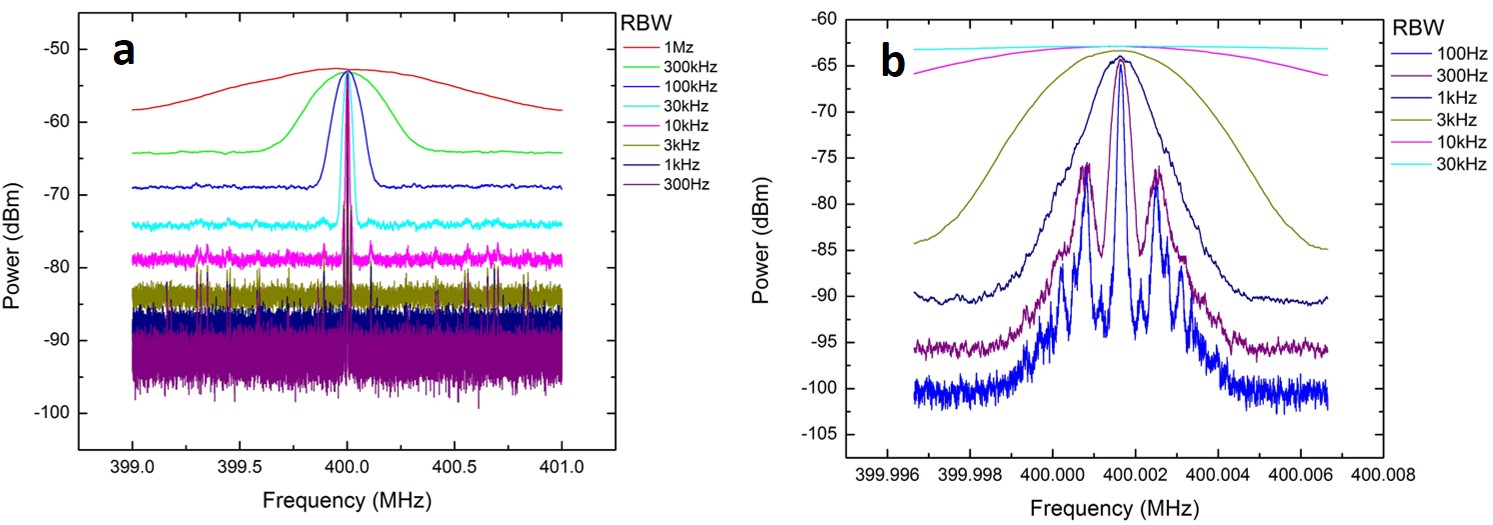


**Supplementary Figure S3.** Intermediate frequency power vs resolution bandwidth. **(a)** Power spectral density at the IF. The peak power does not change significantly for a RBW from 1 MHz to 1 kHz. This means that the optical bandwidth is narrower and, within this RBW range, the signal is not cut out. **(b)** We also observed satellite peaks for the RBW below 1 kHz, that we attribute to the laser instability.

**S4.2. Intermediate frequency power vs wavelength difference between two lasers**

To calibrate the optical power at the IF we used a broadband photoreceiver (New Focus 1554B – 12 GHz). The configuration of the experimental setup is depicted in Figure S4a and is similar to the one used for determining of the conversion bandwidth of the WSSPD. The signal laser is kept fixed at the wavelength of 1550 nm, while the wavelength of the LO is systematically changed. The LO power is kept constant during the wavelength tuning. The measured data are shown in Figure S4b, c. The detectable IF power varies linearly with a slope of 125 MHz/pm. We also found that at the IF power is independent from the frequency difference over a wide range. We measured the working bandwidth, which limits our experimental setup, and within which there is no need to adjust the power of the LO. The cutoff frequency, corresponding to a reduction in power by two *P (IF = 0) / P(f_3dB_) =* 2, is  *f_3dB_* = 5.095 ± 0.25 GHz. This means that within these limit we can measure the conversion bandwidth without any need to adjust the laser power.


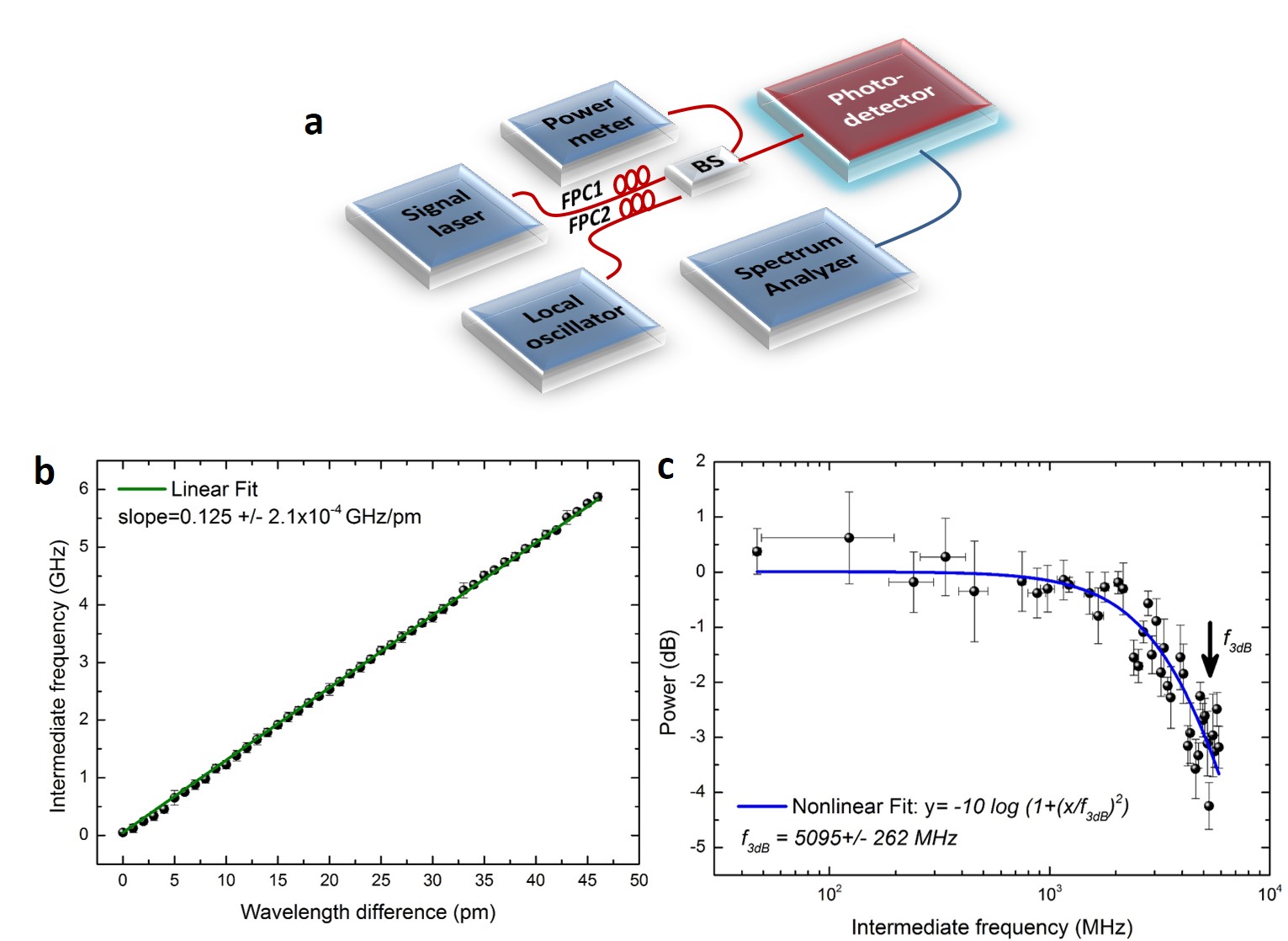


**Supplementary Figure S4.** Calibration of a beating power. **(a)** Schematic view of the experimental setup for the calibration of the beating power. **(b)** Measured dependence of the intermediate frequency *f_IF_* (black circles) vs the wavelength difference between two lasers. The LO (TSL Santec 510) is fixed at the wavelength 1549.966 nm and S (New Focus TLB 6600) is tuned from 1550.015 to 1550.061 nm with 1pm step. The maximum offset difference between two lasers, which can be reached with our setup, is 49 pm. The linear dependence between IF and the wavelength difference has a slope of 125 MHz / pm, close to the theoretically calculated value. **(c)** Measured dependence of the IF Power vs the IF, measured using a wide bandwidth room temperature 12 GHz photoreceiver (New Focus 1554B). From the spectrum, we concluded that the upper frequency limit for the system is around 5.1 GHz, up to which no adjustment of the LO power is needed.

**S5. Estimation of the effective electrical noise**

In addition to the system dark counts *N_SDC_* we have estimated the value of the effective noise *N_eff_* which affects to the SNR for both types of nanowires at measured frequency 400.016 MHz. We used the estimated *N_eff_* in order to compare the data for the ideal detector, compared with the experimentally observed data and make fit.

**S5.1. Estimation of the effective electrical noise from the measurement data**

While comparing the measured SNR with the theory prediction (SE7), one must take into account the contribution of the electrical noise to the measured PSD. To account for this, we introduce an effective dark count number *M_eff_* and rewrite (SE7) as:

$SNR=\frac{M_{S}M_{LO}}{M_{S}+M_{LO}+M_{SDC}+M_{eff}}$. (SE8)

To extrapolate *M_eff_* as well as *N_eff_* = *M_eff_*/*η_OC_* (equation 1 main text), we measured the dependence of the noise PSD on the local oscillator power, expressed in terms of photons per the time 1/RBW. In Figure S5 are presented the result for an intermediate frequency of 400.016 MHz (shown as 400 MHz) and, for comparison, for and IF of 15 MHz. The measured power saturates for a weak photon flux and depends linearly to it at high flux, as expected in case of two independent contributions PSD~ *N_LO_* + *N_eff_*. Our assessment of the effective electrical noise to the U-shaped nanowire for a RBW = 10 MHz gives a value of 2.6, while for W-shaped nanowire *N_eff_* = 700 as indicated in Figure S5b, d. We attribute this difference in the value of *N_eff_* for U-shaped and W-shaped with the fact that on one side the U-shaped nanowire has a shorter pulse duration, on the other hand, the pulse amplitude is less than for W-shaped.

**
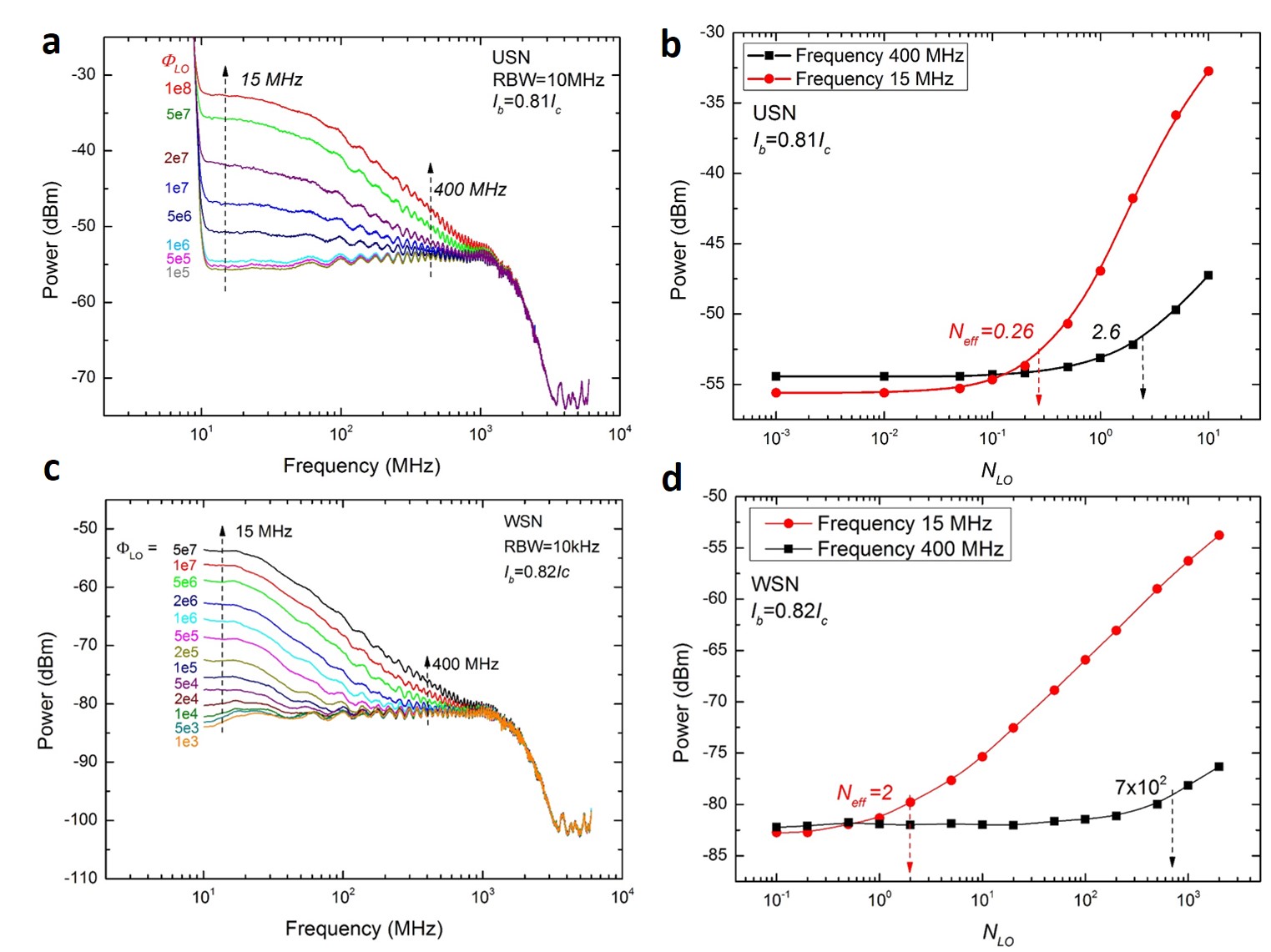
**

**Supplementary Figure S5. Determination of the effective electrical noise (*N_eff_*).** Power vs frequency monitored by the spectrum analyzer for different photon flux, reaching the U-shaped WSSPD (a) and a W-shaped WSSPD (c). The arrows show the power increase for an intermediate frequency of 400MHz and, for comparison, for a frequency of 15 MHz. The dependence of the power vs the photon flux reaching the detector, measured at two different frequencies (400 MHz and 15 MHz) by the SA is depicted in the inset b) for a U-shaped WSSPD and d) for a W-shaped WSSPD. We determine the *N_eff_* as the *N_LO_* at which the power is 3 dB greater than the initial saturated level.

**S5.2. *N_eff_*verification by modeling *SNR* vs *P_LO_***

With decreasing the RBW, the effective contribution of the electrical noise becomes greater:

$N_{eff}(RBW_{2})=N_{eff}(RBW_{1})\frac{RBW_{1}}{RBW_{2}}$ (SE9)

As an example, for a W-shaped nanowire and RBW = 1KHz, the *N_eff_* becomes equal to 7×10^3^ .

In order to show the influence of the *N_eff_* on the SNR we first plot the dependence of both numerator and denominator of the equation SE7 (where *N_eff_* = 0) with respect to *N_S_* (Figure S6a). By dividing the two contributions, one obtains the value of the SNR. In Figure S5b we reported the dependence SNR vs *N_s_* for different *N_LO_* for *N_eff_* = 0.

With the introduction of effective noise, the SNR(*N_s_*) curve varies considerably (Figure S6c). The contribution for different *N_LO_* does not overlap anymore, and the SNR is highly dependent on the local oscillator power. In Figure S6d we fix the signal photons at a value *N_S_* = 10^2^ and plot the dependence SNR vs *N_LO_*. Also in Figure S6d we show a comparison of SNR vs *N_LO_* in absence and in presence of *N_eff_*. It is possible to notice that for *N_eff_* ≠ 0, the SNR depends strongly on the local oscillator power. The same behavior has been observed experimentally (see Figure 5a, main text). **
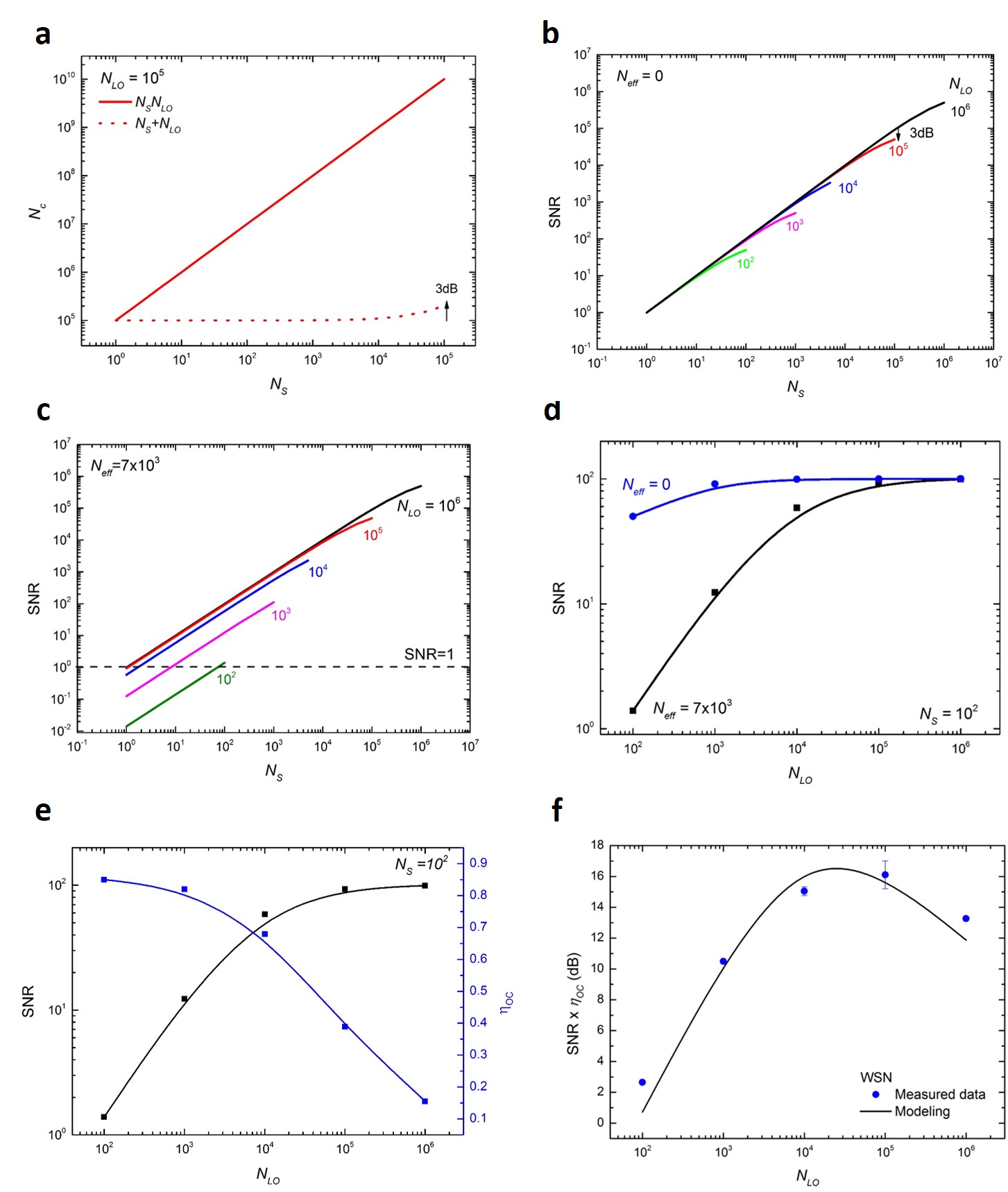
**

**Supplementary Figure S6.** The calculation of SNR according to the formula SE7. **(a)** The dependence of both numerator and denominator on respect to *N_S_* at *N_eff_* = 0*.* **(b)** The SNR vs *N_S_* at different LO powers and *N_eff_* = 0. **(c)** SNR vs *N_S_* for different LO powers and *N_eff_* =7×10^3^. Dashed line indicates SNR equal to unity. **(d)** The dependence of SNR vs *N_LO_* at *N_eff_* = 0 (blue line) and *N_eff_* = 7×10^3^ black line.

**References**

[1 Kerman, A. J., Rosenberg, D., Molnar, R. J. & Dauler, E. A. Readout of superconducting nanowire single-photon detectors at high count rates. *J. Appl. Phys.* **113,** 144511 (2013).

[2] Ferrari, S. *et al.* Waveguide-integrated single- and multi-photon detection at telecom wavelengths using superconducting nanowires. *Appl. Phys. Lett.* **106,** 151101 (2015).

[3] Smirnov, K., Vachtomin, Y., Divochiy, A., Antipov, A. & Goltsman, G. Dependence of dark count rates in superconducting single photon detectors on the filtering effect of standard single mode optical fibers. *Appl. Phys. Express* **8,** 22501 (2015).

[4] Shcherbatenko, M. *et al.* Potential of a superconducting photon counter for heterodyne detection at the telecommunication wavelength. *Opt. Express* **24,** 340–342 (2016).
